# Protocol for mouse vascular dementia model and evaluation of progressive tissue damage in subcortical white matter and adjacent cortex

**DOI:** 10.1016/j.xpro.2026.104396

**Published:** 2026-03-03

**Authors:** Min Tian, Irene L. Llorente, S. Thomas Carmichael

**Affiliations:** 1 Thrust of Bioscience and Biomedical Engineering, The Hong Kong University of Science and Technology (Guangzhou), Guangzhou 511400, China; 2 Department of Neurosurgery, Stanford University, Palo Alto, CA 94304, USA; 3 Department of Neurology, the David Geffen School of Medicine at UCLA, Los Angeles, CA 90095, USA

**Keywords:** Microscopy, Molecular Biology, Neuroscience

## Abstract

Here, we present a protocol for generating a vascular dementia (VaD) mouse model through stereotaxic microinjection of a vasoconstrictor into the subcortical white matter (WM). We describe steps for preparing the N5-(1-iminoethyl)-L-ornithine (L-NIO) solution, surgical procedures, and immunohistochemistry. We then detail procedures for confocal imaging and image analysis using ImageJ and Imaris software to evaluate key pathological features of human VaD, including vessel dilation, demyelination, glial reactivity, neuronal damage, subcortical WM atrophy, and ventriculomegaly.

For complete details on the use and execution of this protocol, please refer to Tian et al.^1^

## Highlights

• Procedures for generating a VaD mouse model via stereotaxic microinjection
• Steps for quantifying core pathological features including cell-type-specific changes
• Instructions for longitudinally tracking pathological progression of disease stages
• Guidance on comprehensive analysis including immunohistochemistry and image processing

## Abstract (editor highlights)

Publisher’s note: Undertaking any experimental protocol requires adherence to local institutional guidelines for laboratory safety and ethics.

## Graphical abstract

**Figure:**
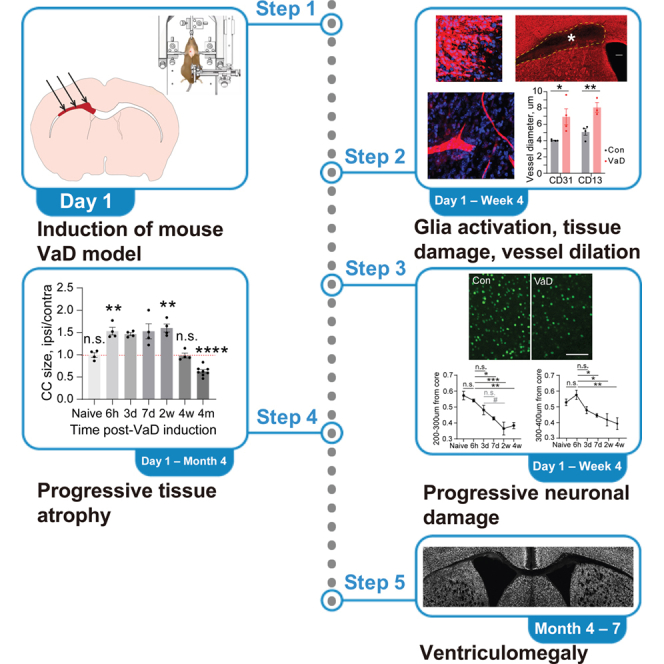

## Before you begin

VaD is the most severe form of vascular cognitive impairment and dementia (VCID).^2^^,^^3^ It accounts for about 25% or higher^4^ of total dementia population and frequently coexists with Alzheimer’s disease (AD). VaD poses distinct challenges for modeling and therapeutic development. It arises from impaired cerebral blood flow^2^^,^^4^^,^^5^ due to cerebrovascular pathologies, including ischemic stroke, microinfarcts, chronic small vessel ischemic disease, and some unknown reasons. Clinically, VaD is diagnosed through cognitive and motor deficits, as well as magnetic resonance imaging (MRI) hyperintensities,^5^ which serve as surrogates for cerebral small vessel disease in subcortical and periventricular white matter (WM).^6^

While rodent models have been instrumental in VaD research, they often fail to fully recapitulate human disease.^7^ For example, bilateral or asymmetrical carotid artery stenosis (BCAS/ACAS) models induce extensive brain hypoperfusion and cause damage in brain regions not commonly affected in patients. The stroke-prone spontaneously hypertensive rat involves complex polygenic mutations and alterations in growth factors and extracellular matrix (ECM) molecules,^8^^,^^9^ complicating molecular studies of VaD. Large mammals such as piglets and primates have been considered excellent for modeling neurological diseases because of their gyrencephalic brain and substantial subcortical WM volume. However, their use has been limited by high cost, limited availability, highly invasive and complex surgical procedures, and suboptimal behavioral readouts, etc.^10^

Recently, a mouse VaD model based on multi-focal ischemia in subcortical WM has shown advantages in mimicking human VaD etiology in various aspects.^1^^,^^11^ Compared with previous models, this model not only recapitulates key human VaD features—including demyelination, axon damage, blood-brain barrier (BBB) leakage, and glial reactivation—but also, for the first time, replicates multiple additional and critical hallmarks of clinal VaD that have never been addressed before. These include localized MRI hyperintensities, disrupted brain circuitry linked to cognitive dysfunction, persistent motor deficits, progressive loss of specific neuronal subpopulations, subcortical WM volume reduction, and ventriculomegaly (enlargement of ventricles). Here, we detail the protocol for inducing this VaD model in C57BL/6 mice^1^ and describe the methods for quantifying and analyzing progressive tissue damages using appropriate software.

### Innovation statement

This manuscript presents a comprehensive and innovative protocol for modeling and investigating vascular dementia (VaD). Its core innovation lies in the introduction and validation of a C57BL/6 mouse model that recapitulates a key diagnostic neuroimaging biomarker of human VaD: **periventricular white matter (WM) ischemia**.

Moving beyond existing models, our approach enables an unprecedented, longitudinal quantification of the disease’s entire pathological cascade. We provide a detailed framework for analyzing dynamic cellular changes and progressive tissue atrophy across a critical, extended time course—from the acute phase (6 h post-induction) to long-term chronic stages (up to 7 months). This protocol offers a unique and powerful platform to deconstruct the spatiotemporal dynamics of VaD progression, thereby accelerating the discovery of mechanisms and therapeutic interventions for a disease notoriously lacking effective treatments.

### Institutional permissions

All animal procedures must be approved by your institution’s IACUC and conform to national guidelines for laboratory animal care.

## Key resources table

**Table:** 

REAGENT or RESOURCE	SOURCE	IDENTIFIER
**Antibodies**		

Rat monoclonal anti-CD13, 1:300 dilution	Abcam	Cat#ab33489; RRID: AB_726095
Goat Polyclonal anti-CD31, 1:300 dilution	R&D	Cat#AF3628-SP; RRID: AB_2161028
Rabbit Polyclonal anti-CD31, 1:300 dilution	Invitrogen	Cat#PA5-16301; RRID: AB_10981955
Rat monoclonal anti-Ctip2, 1:500 dilution	Abcam	Cat#ab18465; RRID: AB_2064130
Rabbit Polyclonal anti-Cux1, 1:500 dilution	Proteintech	Cat#11733-1-AP; RRID: AB_2086995
Rat monoclonal anti-GFAP, 1:1000 dilution	Invitrogen	Cat#13-0300; RRID: AB_2532994
Rabbit Polyclonal anti-Iba1, 1:1000 dilution	Wako	Cat#019-19741; RRID: AB_839504
Goat Polyclonal anti-Iba1, 1:1000 dilution	Abcam	Cat#Ab5076; RRID: AB_2224402
Mouse monoclonal anti-Iba1, 1:1000 dilution	Invitrogen	Cat#MA5-27726; RRID: AB_2735228
Rabbit Polyclonal anti-MBP, 1:500 dilution	Abcam	Cat#Ab40390-1001; RRID: AB_1141521
Mouse monoclonal anti-NeuN, 1:500 dilution	Chemicon	Cat#MAB377; RRID: AB_2298772
Guinea pig Polyclonal anti-NeuN, 1:500 dilution	Synaptic Systems GmbH	Cat#266004; RRID: AB_2619988
Mouse monoclonal anti-NF160, 1:500 dilution	Abcam	Cat#Ab7794-1001; RRID: AB_306083
Rabbit Polyclonal anti-Olig2, 1:500 dilution	EMD Millipore	Cat#AB9610; RRID: AB_570666
Goat Polyclonal anti-Pdgfrβ, 1:500 dilution	R&D	Cat#AF1042; RRID: AB_2162633
Rabbit monoclonal anti-Satb2, 1:1000 dilution	Abcam	Cat#ab92446; RRID: AB_10563678
Fluorophore conjugated Donkey anti-Mouse-IgG (H+L), 1:1000 dilution	Jackson ImmunoResearch	Cat# and RRID depend on type of fluorophore selected
Fluorophore conjugated Donkey anti-Rabbit-IgG (H+L), 1:1000 dilution	Jackson ImmunoResearch	Cat# and RRID depend on type of fluorophore selected

**Chemicals, peptides, and recombinant proteins**		

L-NIO (L-N^5^-(1-Iminoethyl)ornithine dihydrochloride)	Sigma-Aldrich	400600
Mineral oil	Fisher Chemical	O122-1
Isoflurane	Abbott	66794-013-25
10× PBS	Thermo Fisher Scientific	J75889.K8
Triton X-100	MilliporeSigma	TX1568-1
Tissue adhesive	3M Vetbond	1469SB
Super glue	Loctite	N/A
M.O.M. Mouse On Mouse Detection Kit	Vector	BMK-2202
DAPI	Thermo Fisher Scientific	62248

**Experimental models: Organisms/strains**		

C57BL/6 (C57) mice, adult male only	The Jackson Laboratory	JAX: 000664

**Software and algorithms**		

Prism	GraphPad Software	https://www.graphpad.com/features, 8.0 or later versions
ImageJ	National Institutes of Health	https://imagej.nih.gov/ij/index.html, alternatively Fiji can be used
NIS-Elements	Nikon Instruments Inc	https://www.microscope.healthcare.nikon.com, AR4.5.00 or later versions
Imaris	Bitplane	https://imaris.oxinst.com/, 9.0 or later versions

**Other**		

Isoflurane anesthesia setup	VetEquip	RC2 Rodent Circuit Controller, or similar models
Borosilicate glass capillaries	World Precision Instrument (WPI)	1B100F-4
Glass capillary puller	Narishige	model PC-100 Puller (alternative puller can also be used)
Nanoinjector	WPI	2020, alternative can also be used
Nanoinjector controller	WPI	Micro4, or other similar models
Stereotaxic apparatus	Kopf	Model 940, or other models for small animals
Vibratome	Leica	VT1000 S, or other similar models
Confocal microscope	Nikon	C2 or other models/brands

## Materials and equipment

### 1× PBS solution

Dilute 10× PBS with Milli-Q water (v:v = 1:9).

### Anti-freeze solution

To prepare anti-freeze solution, mix the following together:

**Table:** 

Reagent	Final concentration	Amount
1× PBS		500 mL
Sucrose	85.6 g/L	85.6 g
Magnesium Chloride	0.66 g/L	0.66 g
Glycerol		Fill to 1 L

Storage conditions: −20°C, up to 2 years.

0.3% (or 0.5%) TPBS.

To prepare TPBS solution, mix the following together:

**Table:** 

Reagent	Final concentration	Amount
1× PBS		997 mL (or 995 mL)
Triton X-100	0.3% (or 0.5%)	3 mL (or 5 mL)

Storage conditions: 15°C–25°C, up to 1 year if no contamination appears.

• Isoflurane anesthesia setup with induction chamber.
• Stereotaxic apparatus for small animals fitted with digital stereotaxic control panel, and mouse gas anesthesia head holder/nose cone.
• Electric warming pad with controller and rectal temperature probe.
• Pneumatic dental drill with 1/8 Carbide Burr.
• Dissection microscope fitted with a calibrated eye piece reticle.
• Autoclaved surgical tools: fine forceps and fine scissors.
• Sterile cotton tipped applicators.
• 0.9% sterile saline.
• Ophthalmic ointment.
• 70% ethanol.
• Iodine tincture.
• Tissue adhesive.
• Heating pad for postoperative care.
• Nanoliter injector with controller.
• Glass capillary puller.
• Glass pipettes.
• Confocal microscope.
• Vibratome.
• Imaris software.
• ImageJ software.

## Step-by-step method details

### Prepare the L-NIO solution

**Timing: 15 min**

Here, we describe steps for ready-to-use L-NIO solution for intracranial injection.

1. L-NIO-HCL, N5-(1-iminoethyl)-L-ornithine:
  a. Dissolve L-NIO-HCL in sterile 0.9% saline at a concentration of 27 mg/m.
  b. Aliquot and store at −20°C.

**CRITICAL:** Never re-freeze or reuse thawed L-NIO aliquots. Always keep the working aliquot on ice during surgery procedures to avoid deterioration.

### Prepare the nano-injector

**Timing: 15 min**

Here, we describe steps for preparing a nano injector connected to a glass pipet, which is pre-filled with mineral oil and then L-NIO.

2. Pull the glass pipet.

**Figure 1:**
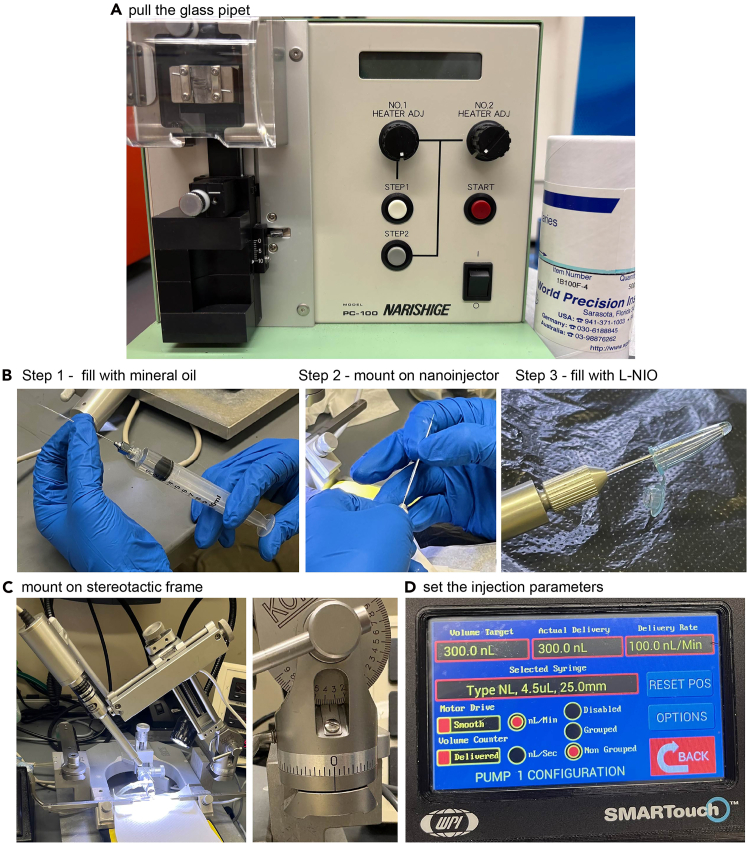
Prepare the glass capillary pipet and nanoinjector (A) Vertical puller and glass capillary. Trim the capillary tip using fine scissors. (B) Use a syringe and long needle to backfill the trimmed glass pipet with mineral oil, mount the syringe onto nanoinjector, and fill the capillary with L-NIO (in a 200 μL tube) using withdraw mode. (C) Mount the nanoinjector onto stereotaxic frame and adjust the angle of nanoinjector to 36°. (D) Set up the injector controller. For L-NIO injection, use syringe: Type NL, 4.5 μL, 25.0 mm; volume target, 300 nL; delivery rate 100.0 nL/Min.

***Note:*** Use borosilicate glass capillaries and glass capillary puller (Figure 1A). While puller settings are model- and condition-dependent, glass capillaries should be pulled to achieve a tip diameter < 10 μm and a taper length of 6–8 mm.

***Note:*** The tip should be trimmed flat with fine scissors to achieve an opening diameter of less than 50 μm.

3. Fill the glass pipet with mineral oil (Figure 1B, step 1).
  a. Mount it onto the nanoinjector (Figure 1B, step 2).
  b. Fill the glass pipet with 2–4 μL L-NIO (Figure 1B, step 3) using withdraw mode at the speed of 20 nL/sec, syringe type, NL, 4.5 μL, 25.0 mm.

***Note:*** To guarantee hydraulic system integrity, it is very important to avoid any air bubbles in the mineral oil and L-NIO (See Problem 4).

4. Mount the prepared nanoinjector onto the stereotaxic frame with a 36° angle (Figure 1C).
5. Set the controller (Figure 1D): volume, 300 nL; delivery rate, 100 nL/min; syringe type, NL, 4.5 μL, 25.0 mm.

### Anesthetize the mouse and mount on a stereotaxic frame

**Timing: 5 min**

Here, we describe the pre-surgical preparation steps before intracranial surgery in VaD model.

6. Check the tubing of rodent circuit controller are well connected with the induction chamber and the stereotaxic frame.
7. Transfer a mouse from its home cage to the induction chamber (Figure 2A).

**Figure 2:**
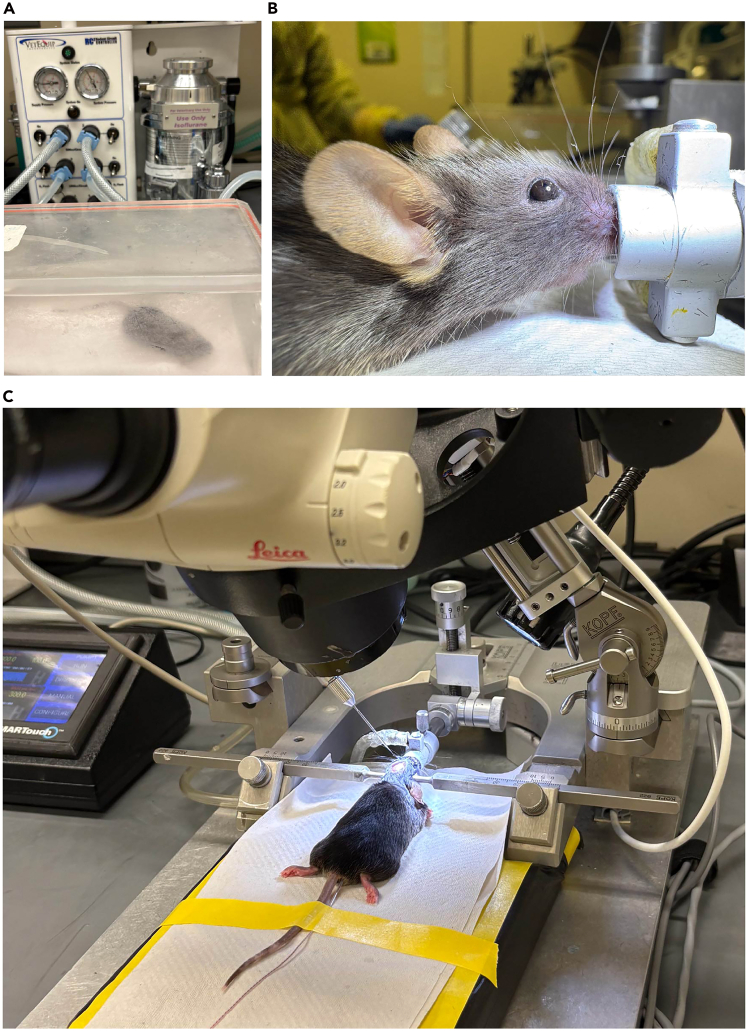
Anesthetizing the mouse and securing its head in a stereotaxic frame (A) VetEquip connected with an induction chamber is used to induce anesthesia of C57BL/6 mice. (B) The mouse is positioned to a stereotaxic frame with an isoflurane nose cone. (C) Place a heat pad under the mouse body and monitor the rectal temperature using a thermal probe and heat pad controller. The mouse skull is exposed after hair removal and skin opening. Position the glass pipet on top of the bregma under a surgical microscopy.
8. Set the induction chamber (1–2 L) to deliver 4% isoflurane with an oxygen flow rate of 2–3 L/min and initiate the flow.
9. Wait until the breath rate of the mouse is about 1 time per second.

**CRITICAL:** Check for full anesthesia by testing the interdigital reflex on the paw.

10. Turn the isoflurane to 2% and oxygen flow to 0.5–1.0 L/min, and turn on the connection towards the stereotaxic frame.
11. Turn on the heat pad.
12. Position the mouse to stereotaxic frame with an isoflurane nose cone (Figure 2B).
13. Insert the incisor adapter into the mouse’s mouth until its incisors fit into the small opening in the adapter.

**CRITICAL:** Make sure the tongue is not blocking the breathing path.

***Note:*** Slightly pull back the incisor adapter to check for its correct position.

14. Fix the incisor adapter and carefully place the snout clamp just below the eyes using low pressure.
15. Place the rectal thermal probe to monitor the mouse body temperature (Figure 2C).

**CRITICAL:** A good body temperature for VaD induction is 36.5°C–37.5°C.

16. Fit the ear bars to the lateral side of mouse skull (caudal side of squamosal sutures and rostral side of the ears) (Figure 2C).
17. Apply lubricant eye ointment to prevent dryness of the eyes.
18. At this point, the mouse’s head should be completely fixed in the stereotaxic apparatus and no movement should be possible.

### Induction of subcortical WM ischemia in mouse

**Timing: 45 min**

Here, we describe the surgical steps for VaD mouse model based on subcortical ischemia.

19. Remove the scalp hair with a trimmer to expose the surgical site. Alternatively, hair remover cream can be used.
20. Remove the hair residue using 70% ethanol and cotton tipped applicators.
21. Disinfect the head skin with iodine tincture followed by 70% ethanol.
22. Make a midline scalp incision (1 cm-long) using scissors or a thin scalpel on the head skin.
23. Expose the most caudal part of olfactory bulbs, bregma and lambda, and the most lateral side of left skull.
24. Scrab the skull with cotton tipped applicator to remove any soft tissue on the skull.

***Note:*** The cotton tipped applicator can also dry the skull and help better visualize the cranial sutures.

**Figure 3:**
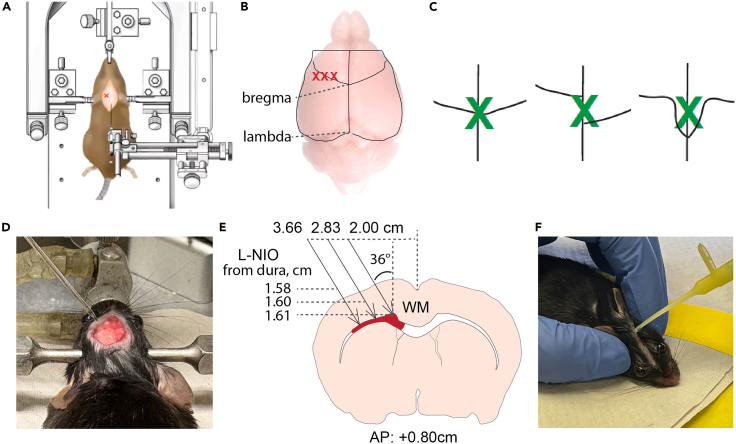
Intracranial injection of L-NIO into mouse subcortical WM (A) A diagram showing the locating the coordinates using a stereotaxic frame and glass pipet. (B) A diagram showing the location of three injection sites relative to bregma and lambda. (C) Identification bregma in different skull suture conditions. Green cross indicates the location of bregma. (D) An image showing the glass pipet inserted into mouse brain, ready for L-NIO injection. (E) A schematic showing the coordinates and angle of three injections into subcortical WM in mouse brain. (F) After injections, close the scalp using tissue adhesive.
26. Visualize the skull sutures through a dissection microscope using 20× to 40× magnification and identify exact position of bregma (Figures 3B and 3C).
27. Move the capillary to bregma and slightly touch the bregma with the tip of the capillary. Set AP (anterior-posterior), ML (medial-lateral) and DV (dorsal-ventral) coordinates to 0 in the stereotaxic.
28. Move the capillary left and right along the ML axis.
  a. Adjust the height of capillary along the DV axis to avoid breaking the capillary.
  b. Slightly touch the skull at coordinates: AP +0.00 mm, ML +2.00 mm; and AP +0.00 mm, ML -2.00 mm.

***Note:*** This step is to ensure that the left and right cerebral hemispheres are aligned on the same horizontal plane.

25. Adjust the height of the incisor adaptor to make sure the bregma and lambda are on the same horizontal plane (Z coordinates difference within ±50 μm), and the midline of brain suture align with the midline of stereotaxic frame (Figure 3A).

**CRITICAL:** If a bilateral DV coordinate deviation exceeding ±50 μm is observed, realign the ear bars until symmetrical DV coordinates are achieved.

29. Adjust the angle of nanoinjector to 36° in order to minimize damage in motor cortex.
30. Drill bur holes on the skull with following AP and ML coordinates targeting subcortical WM.
  ***Note:*** ML coordinates:
  AP +0.80 mm, ML +2.00 mm, DV −1.56 mm from dura.
  AP +0.80 mm, ML +2.83 mm, DV −1.60 mm from dura.
  AP +0.80 mm, ML +3.66 mm, DV −1.61 mm from dura.
  a. Clear the surgical site by gently removing any bone debris and blood with saline-moistened cotton swabs.
    **CRITICAL:** Position the capillary tip by slowly lowering it until it just touches the dura mater, then set this position as DV = 0.00 mm.
  b. To prevent capillary occlusion prior to injection, position the tip approximately 1.00 mm above the dura.
  c. Deliver 30–50 μL of L-NIO until a droplet form at the tip.
    **CRITICAL:** Absorb any excess solution with a Kimwipe before advancing the capillary to the final DV coordinate for injection.
  d. Place capillary to desired DV coordinate, allow a 5-minute pre-infusion period.
  e. Deliver 0.3 μL of 27 μg/μL L-NIO (in sterile physiological saline) at each target site using an infusion rate of 100 nL/min (Figures 3D and 3E).
    **CRITICAL:** Upon completion of the infusion, maintain the capillary in place for an additional 5 min before slow withdrawal.
    ***Note:*** Coordinates may vary between C57BL/6 mice from different vendors, with different ages and sexes, and even experimenters. Pilot experiment to optimize the coordinates can be necessary.
31. Close the scalp with tissue adhesive (Figure 3F).
32. Allow the mouse to recover in home cage with half bottom on an electric heat pad at 37°C. Provide free access to food and water.
33. After all the mice in a cage are recovered from surgery, change the water bottle to Sulfamethoxazole and Trimethoprim Suspension (TMS, v:v = 1:100 dilution in drinking water) for 7 days to avoid any infections after surgery.

**CRITICAL:** While antibiotics after surgery may not be requested under certain circumstances, anti-inflammation medicines, such as NSAIDs (non-steroidal anti-inflammatory drugs), must NOT be used because they will affect the recovery of infarct lesion.

34. Monitor the mouse at 24 h after the surgery by checking the wound and its weight.

***Note:*** If the scalp is open, anesthetize the mouse again, clean the wound, and re-suture the opening with tissue adhesive.

35. Further monitor the mice for 7–10 days.

### Perfusion and vibratome sectioning (post-injection day 7, 14, 28, or depending on the aim of studies)

**Timing: 2 days (2 h hands-on)**

Here, we detail the steps for collecting and sectioning brain tissue for immunostaining in order to analyze lesion progression.

Day 1.

36. Anesthetize the mouse in an isoflurane chamber.
37. Put a nose cone with isoflurane to make sure the mouse kept anesthetized. Secure the mouse in a supine position on a styrene platform by affixing its paws with fine-gauge needles.
38. Expose the heart, insert venofix needle into the left ventricle and open the right atrium.
39. Perfuse the mouse with a peristaltic pump with 20 mL of 1× PBS followed by 20 mL of 4% PFA at a rate of 2 mL/min.
40. Cut the head using big scissors and open the scalp.
  a. Using fine forceps and scissors, gently cut and remove the top of skull.
  b. Cut the optic nerves.
  c. Transfer the brain into a 50 mL falcon tube filled with 10 mL of 4% PFA.
41. Gently shake the tube with brain and PFA for further fixation for 12–36 h in 4°C.

Day 2.

42. Change the fixing 4% PFA solution into 1× PBS.

**CRITICAL:** Section the brain within 7 days to avoid possible degradation of target proteins.

43. Cut the cerebellum flat and glue (super glue) the brains vertically on the stage of vibratome with olfactory buds facing upward.
44. Place and secure a shaving blade on blade holder.
45. Fill the sectioning tank with 1× PBS and merge the brain samples and cutting blade.
46. Set the vibratome for a sectioning thickness of 30–50 μm, a blade amplitude of 0.6–1.2 mm, and a speed of 0.6–1.2 mm/sec.

***Note:*** These parameters are starting recommendations and should be optimized for your specific experiment and equipment.

47. Set start and end points.
48. Cut 50 μm (recommended) coronal brain sections. Collect the sections from the prefrontal cortex to hippocampus along the AP axis.
49. Collect the sections in a 24-well-plate filled with the anti-freeze solution. Collect 6 consecutive sections per well so that each well is separated by 300 μm.

***Note:*** Approximately 18 wells will be used for all the sections, covering brain regions from prefrontal cortex to ventral hippocampus along the AP axis.

50. After finishing the sectioning, label and wrap the plate using aluminum foil, store in −20°C before use.

### Immunohistochemistry

**Timing: 2 days (adjustable if different antibodies are used which require retrieval and/or longer incubation time)**

Here, we describe the immunostaining protocol used to analyze the progressive pathological changes in mouse VaD model.

***Note:*** In this part, we describe immunohistochemical staining targeting the following antigens, either individually or in combination: NF160+ (axon projections), MBP+ (myelin), Iba1+ (microglia/macrophages), GFAP+ (astrocytes), CD31+ (endothelial cells), CD13+ (pericytes and fibroblasts), Pdgfrβ+ (pericytes), Olig2+ (oligodendrocyte lineage cells), NeuN+ (neurons), and subpopulation of neurons including Satb2+, Cux1+, and Ctip2+ cells.

51. Observe under a dissection microscope to find the wells that contain sections with ischemic lesion.
52. Use angled forceps to gently transfer the sections to a 24-well-plate filled with 1× PBS.

***Note:*** Maximum 3 slices per well.

53. Wash the sections with 1× PBS, 3× 5min.

***Note:*** When using pipette tips to remove or add liquid, avoid direct contact with the tissue slices. If transferring slices between wells, use a soft brush to minimize mechanical damage.

54. Block and permeabilize the sections with blocking buffer (5% normal donkey serum in 0.5% Triton X in 1× PBS) for 1 h at 15°C–25°C.

***Note:*** For staining of NF160 antibody, or any mouse host antibody, will be required the use of M.O.M. in blocking buffer (v:v = 1:40).

55. Incubate with primary antibody in 5% normal donkey serum in 0.3% TPBS for 12-36 h at 4°C. Extend the incubation time to 48 h if necessary.
56. Wash the sections with 1x PBS, 3x 5 min.
57. Prepare secondary antibody solutions (fluorophore conjugated donkey IgG) in 3% normal donkey serum in 0.3% TPBS (v:v = 1:1,000).

**CRITICAL:** Make sure the wavelength of fluorophores for co-stained antibodies of different species does not overlap.

58. Incubate with secondary antibody solution for 1 h at 15°C–25°C.
59. Wash the sections with 1× PBS, 2× 5min.
60. Counterstain all sections with DAPI (dilute with 1× PBS, v:v = 1:500) for 5 min at 15°C–25°C.
61. Wash the sections with 1× PBS, 2× 5min.
62. Mount sections with 50% glycerol and seal the coverslip with nail polish.

***Note:*** If other type of mounting medium is preferred, follow the product instructions.

63. Airdry in dark for 12–36 h and keep at 4°C until imaging.

### Confocal imaging

Here, we describe how to acquire high-resolution z-stack images with Nikon C2 confocal microscope.

***Note:*** Four channel acquisition (408, 488, 546, and 647 nm) is preferred.

**CRITICAL:** Use the identical pixel size, pinhole size, excitation and detection settings, step-size and number of z-stacks, and other scan parameters across all acquisitions and all groups for the same set of experiments.

64. Use 10× or 20× objectives to view and adjust the focus on the lesion region.
65. Scan the section to set appropriate excitation intensity and detection gain for each channel, so that the emission signals are not too low or saturated.
66. Save the optimal settings for batch scans.

***Note:*** If large scan is preferred, use the same number of tiles (x by y) for all the sections in the same experiment.

67. Stacked or single z panel .tiff files can be used for quantification in ImageJ, and .nd2 (if Nikon confocal is used) files can be converted into .ims files for quantification analysis in Imaris.

### Image quantification

Here, we describe the quantitative analysis procedures used to assess progressive lesion changes in a mouse VaD model, based on images acquired in the preceding steps.

***Note:*** As specified below, use Imaris for 3D reconstruction and advanced quantification; use ImageJ for 2D measurement;

68. In Imaris, utilize the “Surfaces” function to segment volumes or the “Spots” function to identify and count punctate structures.
  a. Apply identical thresholds across all experimental groups to ensure unbiased comparison.
  b. Normalize all quantitative outputs as described in each subsection.

**CRITICAL:** We recommend quantifying 4–8 animals for each experimental condition, and 4 slices with 300 μm interval across the lesion core along the AP axis per animal. Quantify across multiple time points to track progressive degeneration.

69. Quantification of core size, axon volume and its myelination using Imaris.
  a. Use the coronal brain sections stained with MBP, NF160 and DAPI.
  b. In Imaris, use surface function (Figure 4A) to manually draw the regions of ischemic lesion core which is defined by aggregation of DAPI+ particles, axonal damage (NF160) and MBP debris (MBP) (Figure 4D).
    ***Note:*** The value of core size will appear when the analysis result is exported. The same for value of axon volume (iv), and myelination (v) in following Imaris analyses.

**Figure 4:**
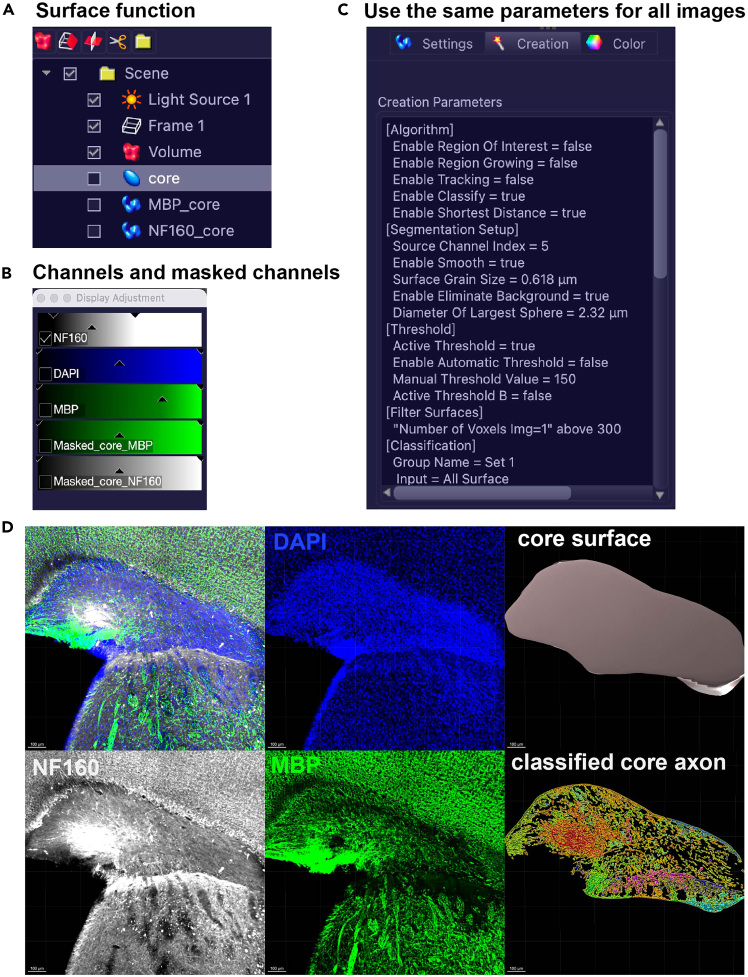
Surface function in Imaris for quantification of core size, axon volume and its myelination (A) Create and name the surfaces for core, MBP_core and NF160_core. (B) Masking the core_MBP and core_NF160 channels. (C) A list of parameters for analyzing MBP or NF160 surfaces under “creation” icon. Make sure all the images are analyzed using the same series of parameters. (D) Representative images showing (left to right, top to bottom): Co-staining of NF160 (white), MBP (green), and DAPI (blue) in subcortical WM ischemic lesion; aggregation of nuclei in the lesion core shown by DAPI staining; Core surface; breakdown and debris of neurofilament shown by NF160 staining; debris of myelination damage shown by MBP staining; core axons classified by the extent of myelination.
  c. Mask the MBP and NF160 channels and name them as Masked_core_MBP and Masked_core_NF160 (Figure 4B).
  d. Setup the parameters for axon quantification, i.e., source channel = Masked_core_NF160, surface grain size, diameter of largest sphere, threshold value, filters and values, classification of result surfaces, etc (Figure 4C).
  e. To quantify the extent of myelination of axons, classify the surfaces by ”Overlapped Volume Ratio to Surfaces = MBP_core”: < 0.1, 0.1–0.2, …0.9–1.0 (Figure 4D, lower right).
70. Progressive myelin and axon volume loss.
  a. Euthanize the VaD animals at different time points (6 h to several months) after L-NIO injection. Use the coronal brain sections stained with MBP and NF160.
  b. In Imaris, use surface function to draw the regions of ipsilateral to contralateral subcortical WM, quantify the volume of both regions (V_ipsi and V_contra).
  c. Mask the MBP and NF160 channels and name them as MBP_wm_ipsi and MBP_wm_contra, NF160_wm_ipsi and NF160_wm_contra respectively. Set threshold and quantify the volume of each channel (V_MBP_ipsi and V_MBP_contra, V_NF160_ipsi and V_NF160_contra).
    i. Quantification of MBP or NF160 volume changes.
      ***Note:*** Because the absolute volume of subcortical WM varies between coronal slices depending on the coordinate along AP axis, it is difficult to directly compare the absolute volume of NF160 or MBP between groups.
      ***Note:*** The volume of ipsilateral and contralateral subcortical WM is very similar to each other. Therefore, we first normalize the ipsilateral NF160/MBP volume to the contralateral hemisphere (which served as an internal control), and then compare the normalized values (ranging from 0 to 1) between groups. We think this is a more reasonable way to investigate how the axon reprojection and myelin regrowth is changed by treatment.
      ***Note:*** The change of MBP volume is calculated as: sample (V_MBP_ipsi/V_ipsi)/(V_MBP_contra/V_contra) – naïve control (V_MBP_ipsi/V_ipsi)/(V_MBP_contra/V_contra).
      The change of NF160 can be calculated in the same way.
    ii. Quantification of myelinated NF160 in lesion core as a readout of progressive change.
      ***Note:*** One of the progressive natures in VaD mouse model is that axon processes in the lesion core and adjacent WM are demyelinated in acute and subacute stages, which gradually retract or be remyelinated in chronic stages. Therefore, the percentage of myelinated axon volume can be used as a marker for VaD progression in longitudinal study.
      ***Note:*** We can use NF160 and MBP co-staining to quantify this process, i.e. more NF160+ neurofilaments are not overlapped with MBP in acute and subacute stages, while most of NF160+ signal overlap with MBP+ in chronic stages.
      In specific, euthanize animals at different timepoints after VaD; co-stain coronal brain sections with NF160, MBP, DAPI and GFAP (optional); image with confocal microscopy. In Imaris, follow the steps in 1, and quantify the myelination of core axons (NF160) in samples euthanized at different timepoints after VaD surgery.
71. Lesion core size measurement using ImageJ.
  ***Note:*** While Imaris is good for quantification of lesion core size (see A above), ImageJ is also an appropriate and a free alternative for core size quantification.
  a. Co-stain VaD brain sections with DAPI, NF160, MBP and GFAP (optional). Image the slides using confocal or epifluorescence microscopy (10× or 20×) and export .tiff files for each sample (Figure 5A, adopted from Tian, et al^1^).

**Figure 5:**
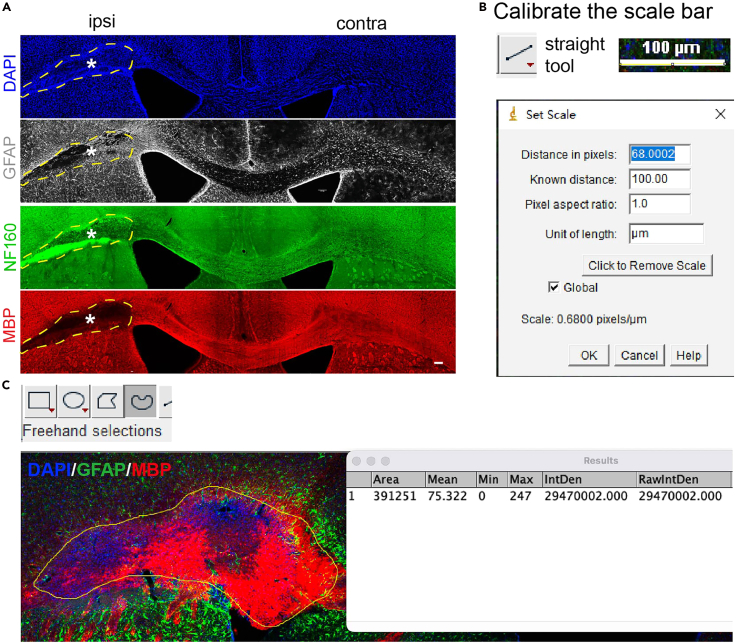
Quantification of lesion size using ImageJ (A) Drawing the lesion border indicated by GFAP staining (grey), damage of neurofilaments (NF160, green), and demyelination (MBP, red). Asterisk, lesion core; dashed line, core margin, scale bar = 100 μm. Adopted from Tian, et al.^1^ (B) Calibrating the scale bar in ImageJ analysis using straight tool, pixel/μm. (C) Selection of ROI (yellow line) and measurement of area and intensity using Freehand selection.
  b. In ImageJ, calibrate the scale bar to set the number of pixel per μm (Figure 5B).
  c. Use freehand tool to draw the lesion core border, which is defined by DAPI aggregation, NF160/MBP debris, and GFAP barrier.
  d. Measure the absolute size of lesion core (μm^2^) (Figure 5C).
72. Brain atrophy.
  ***Note:*** Two important biomarkers of VaD progression are the shrinking periventricular WM and enlarging ventricles (ventriculomegaly). Our mouse VaD model replicates these processes from subacute to chronic stages after surgery, which can be quantified using immunostaining and ImageJ analysis.
  a. Co-stain VaD brain sections with DAPI and NeuN.
  b. Image the slides using confocal or epifluorescence microscopy (10× or 20×) and export .tiff files for each sample.
    ***Note:*** Subcortical WM is featured by adjacency to lateral ventricles, with no NeuN+ cells.
  c. In ImageJ, calibrate the scale bar to set the number of μm per pixel.
  d. Quantification of subcortical WM size.
    i. In ImageJ, use freehand tool to draw the boundary of ipsi- and contra-lateral WM (Figure 6, blue area) and read the size(s) in μm^2^.

**Figure 6:**
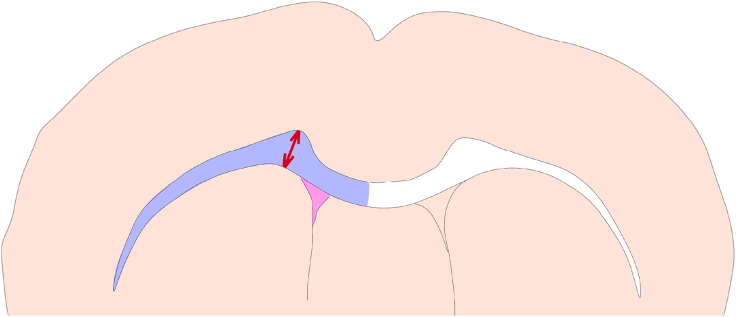
Schematic diagram illustrating the anatomical boundary of the subcortical WM (blue), its thickness (red double-headed arrow), and the border of the lateral ventricle (pink) at one side of mouse brain
    ii. The change of WM size caused by ischemic lesion in VaD is defined as the ratio of ipsilateral to contralateral WM.
      ***Note:*** If the ratio value is 0–1.0, the ipsilateral WM is shrinking; if the ratio value is 1.0, no change; if the ration value is > 1.0, the ipsilateral WM is enlarged, probably due to edema caused by blood-brain barrier (BBB) leakage in acute and subacute stages.
    iii. Compare the change across time.
  e. Quantification of subcortical WM thickness.
    i. In ImageJ, use the straight tool to draw a line covering the thickest part of WM (Figure 6, red double-headed arrow) where the lesion usually occurs.
    ii. Measure the length of the line (μm) at ipsilateral and contralateral hemisphere. The change of WM thickness is defined as Thickness_ipsi/Thickness_contra.
    iii. Compare the change across time.
  f. Quantification of ventricle size.
    i. In ImageJ, use the freehand tool to draw borders along the margin of ipsi- and contra-lateral ventricles (Figure 6, pink area).
    ii. Measure the sizes (μm^2^). The change of ventricle size is defined as Size_ipsi/Size_contra.
      ***Note:*** If the ratio value is 0–1.0, the ipsilateral ventricle is shrinking which might be caused by edema in ipsilateral WM in acute and subacute stages; if the ratio value is 1.0, no change; if the ration value is > 1.0, the ventricle is enlarged (ventriculomegaly) which might be caused by shrinkage of WM in subacute and chronic stages.
    iii. Compare the change across time.
73. Progressive loss of neuronal subtypes.
  ***Note:*** An important marker for progressive change in VaD mouse model is that the transcriptional factors in the distal layer VI, 200–400 μm away from ischemic lesion, are gradually reduced along time.^1^
  a. To quantify the change, co-stain transcriptional factor(s) Satb2/Cux1 with pan-neuronal marker NeuN and nucleus marker DAPI (Figure 7A).

**Figure 7:**
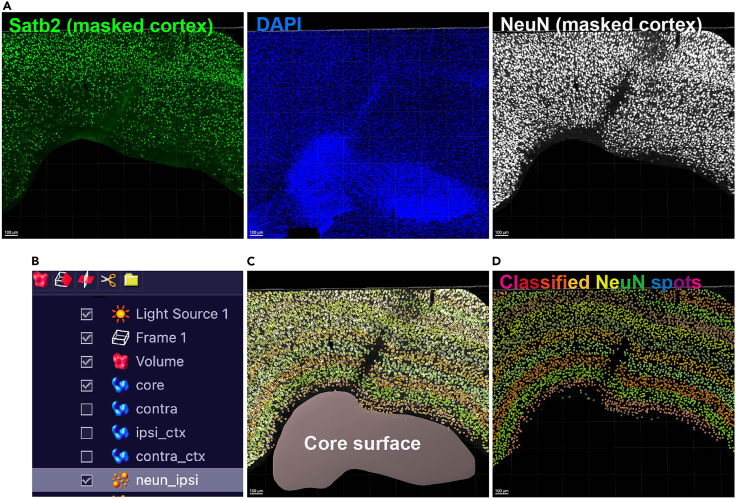
Quantification and classification of neuron subtypes in lesion adjacent cortex in Imaris (A) (Left) Masking Satb2 (green) channel in lesion adjacent cortex. (Middle) Aggregation of nucleus (DAPI+, blue) in ischemic lesion core in VaD mouse brain. (Right) Masking NeuN (white) channel in lesion adjacent cortex. (B) Creation of surfaces for core and ipsi_ctx (ipsi lateral cortex), spots for neun_ipsi, etc. (C) Combined core surface, masked NeuN+ channel in lesion adjacent cortex, and spot classification of NeuN+ cells by distance away from core. (D) Spot classification of NeuN+ cells by distance away from core. Scale bars = 100 μm.
  b. Image the slides using confocal microscopy and analyze the .nd2 files (if Nikon used) using Imaris. In specific, create surfaces for core and cortex in the ipsilateral hemisphere by manually drawing the margins (Figure 7B).
  c. Mask the Satb2/Cux1, NeuN and DAPI channels in cortex.
  d. Quantify the number of Satb2+ cells in cortex using spots function, and classify the spots by the distance to core: 0–100 μm, 100–200 μm, etc.
  e. Repeat the spot quantification process for Cux1, NeuN (Figures 7C and 7D) and DAPI. The change of Satb2+ neural subtypes is analyzed by comparing the Satb2/NeuN or Satb2/DAPI value at the same distance a long time.
74. Glial and vascular responses.
  a. Activation of microglia.
    i. Stain the VaD and control brains with Iba1 antibody.
    ii. Image the slides using confocal microscopy and export .tiff files.
    iii. In ImageJ, use freehand tool to draw boundary of subcortical WM as the region of interest (ROI), measure the average intensity in Iba1 channel.
    iv. The change of Iba1 immunoreactivity is defined as the ratio of Average intensity of Iba1 channel in VaD brain over control brain.
  b. Dilation of blood vessels (mostly capillaries in subcortical WM)
    ***Note:*** Ischemic injury in VaD disrupts endothelial cell tight junctions, resulting in vasodilation.
    i. To visualize and quantify the vessel dilation, stain the VaD and control brains with CD31 or CD13 antibodies.
    ii. Image the slides using confocal microscopy and export .tiff files.
    iii. In ImageJ, use straight tool to draw lines across the diameter of vessels, read the length of the line.
    iv. Randomly measure 3–5 blood vessels in each image/ROI and average all the readings.
    v. Vessel diameter change is quantified as the ratio of the average vessel diameter in VaD model brains to that in control brains.
  c. Activation of pericyte/fibroblast.
    ***Note:*** Pericytes play diverse roles during injury, including angiogenesis,^12^ ECM component,^13^ as well as formation of fibroblasts.^14^ Specific pericyte markers that allow for unambiguous discrimination from fibroblasts have not been definitively established.
    ***Note:*** While CD13 and Pdgfrβ are common pericyte markers, they also cross-react with fibroblasts. Conversely, CSPG4 (NG2) labels pericytes but is not specific, as it is also expressed by oligodendrocyte lineage cells.
    ***Note:*** Here we use CD13 to investigate the activation of pericytes and fibroblasts in the VaD lesion core.
    i. Stain the VaD and control brains with CD13 antibody.
    ii. Image the slides using confocal microscopy and export .tiff files.
    iii. In ImageJ, use freehand tool to draw boundary of subcortical WM as the region of interest (ROI), measure the average intensity in CD13 channel.
    iv. The change of CD13 immunoreactivity is defined as the ratio of Average intensity of CD13 channel in VaD over control.
  d. Activation of OPCs.
    ***Note:*** During injury, differentiated oligodendrocytes (OL) die while OPCs can be activated and their proliferation can be increased. Using the oligodendrocyte lineage marker Olig2 can demonstrate the change in the OL population, which is majorly contributed by proliferation of OPCs.
    i. Stain the VaD and control brains with Olig2 antibody.
    ii. Image the slides using confocal microscopy and export .nd2 files (if Nikon is used).
      ***Note:*** In Imaris, transform .nd2 files to .ims files and analyze using surfaces and spots function. In specific, create surfaces for the ischemic subcortical WM in the VaD mouse brains and the homologous region of control brains by manual drawing the margins. Read the value of surfaces.
    iii. Mask the Olig2 channel.
    iv. Create a spot and quantify the number of Olig2+ cells in the masked Olig2 channels.
    v. Normalize the number of Olig2+ cells with corresponding surface values (number of cells/mm^3^).
    vi. The change of Olig2+ cells is calculated by comparing VaD brains to control brains.
  e. Astrocyte activation and formation of the reactive barrier.
    i. Stain the VaD and control brains with GFAP antibody.
    ii. Image the slides using confocal microscopy and export .tiff files.
    iii. In ImageJ, use freehand tool to draw boundary of subcortical WM as the region of interest (ROI).
    iv. Measure the average intensity in GFAP channel.
    v. The change of GFAP immunoreactivity is defined as the ratio of Average intensity in VaD brain over control brain.

## Expected outcomes

Based on the findings from Tian et al.,^1^ the following pathological and functional outcomes are anticipated in this vascular dementia (VaD) mouse model.

During the acute and subacute stages (less than 4 weeks post-surgery), subcortical white matter (WM) is expected to exhibit significant axon and myelin damage, blood-brain barrier leakage, and activation of key glial and vascular cell populations, including microglia, oligodendrocyte precursor cells, pericytes or fibroblasts, and astrocytes. Dilation of blood vessels in subcortical WM in and near ischemic lesion is also anticipated. At a cellular level, a progressive reduction in transcriptional factors (Satb2 and Cux1) is expected in the distal cortical layer VI. Structurally, the ipsilateral WM may appear enlarged, potentially due to post-ischemic edema. These structural changes will be accompanied by measurable memory, memory linking, and motor deficits.

In the chronic stage (beyond 4 weeks post-surgery), the initial WM enlargement is expected to reverse, progressing to a shrinkage due to the established ischemic lesion. Concurrently, the ventricle in the ipsilateral hemisphere becomes enlarged (ventriculomegaly). Critically, both memory and motor deficits are expected to persist throughout this chronic phase.

## Limitations

This VaD model exhibits several inherent limitations. First, inaccuracies in the injection angle or depth may result in missing the target subcortical white matter (WM), potentially depositing L-NIO into the cortex or striatum, and can also cause minor variations in lesion volume. Second, the model is influenced by estrogen-mediated protection, which precludes the straightforward use of young adult female mice due to fluctuating ischemic damage across the estrous cycle; employing females would necessitate a significantly larger sample size to account for this variability. Finally, while the model recapitulates a key aspect of human VaD—focal subcortical WM ischemia—it does not fully emulate the etiological or genetic complexity of the human condition. Notably, it lacks other pathological features commonly found within the spectrum of human ischemic WM disease, such as microhemorrhages, vascular amyloid deposition, or small vessel arteriopathy.^15^

## Troubleshooting

### Problem 1

Infections after surgery.

### Potential solution

An antibiotic is required post-surgery; we have found that including cherry-flavored TMS in the drinking water as opposed to less-sweet antibiotics promotes increased consumption by the mice and therefore better recovery and rehydration.

TMS is light sensitive and should be protected from light in amber-or red-colored water bottles. Alternatively, water bottles can be wrapped in aluminum foil, although mice will tend to scratch away the foil.

### Problem 2

Big needle track in motor cortex.

### Potential solution

Use short-tapered glass pipette. Adjust the temperature and strength of capillary puller in order to pull a long-tapered micropipette (>4 mm is preferred).

### Problem 3

Missing cerebral white mater during L-NIO injection.

### Potential solution

Adjust coordinates. Coordinates may vary between C57BL/6 mice from different vendors, with different ages and sexes, and even experimenters. Pilot experiment to optimize the coordinates can be necessary.

### Problem 4

Inconsistent lesion size.

### Potential solution

Inconsistent lesion size can arise from multiple technical and biological variables. The following factors should be systematically checked to ensure reproducibility.

1. **Stereotaxic Accuracy:** Verify the angle and depth of the glass capillary before injection. Even minor deviations in stereotaxic settings can lead to significant variability in lesion location and size.
2. **Hydraulic System Integrity:** Ensure no air bubbles are present in the mineral oil or L-NIO line, as compressible air bubbles directly affect injection volume accuracy. To avoid introducing air bubbles, carefully adhere to the procedure outlined in Figure 1B: Insert a mineral oil-filled syringe into the tip of the glass pipette, then gently advance the plunger while slowly withdrawing the syringe barrel. Continue until oil droplets emerge from the pipette tip and the pipette is completely filled with oil. Next, fill the pipette with L-NIO using a speed no faster than 20 nL/sec.
3. **Injection Volume Calibration:** Confirm that the correct syringe type is selected in the nanoinjector controller software. Regular calibration is essential for precise volume delivery.
4. **Drug (L-NIO) Stability:** Aliquot L-NIO to avoid repeated freeze-thaw cycles, which degrade its efficacy. The working aliquot must be kept on ice throughout the surgery, and any remaining solution should be discarded post-procedure.
5. **Pipette Seal and Patency:** After assembling a glass pipette, verify a gas-tight seal by testing the injection function. Visually confirm fluid ejection and validate volume accuracy by collecting 1 μL on parafilm for measurement.
6. **Pipette Tip Clogging:** The tip may be clogged by blood or bone debris during craniotomy. Gently clear the tip by tapping it on a saline-moistened Kimwipe. Before injection, expel ∼50 nL of L-NIO to ensure patency, then dry the tip.
7. **Leakage During Withdrawal:** Although uncommon with glass capillaries in subcortical white matter (WM), leakage can occur. If observed, allow the pipette to rest in place for 5 min post-injection before withdrawing slowly to minimize backflow.
8. **Animal Body Temperature:** Maintain core body temperature at 36.5°C––37.5°C throughout the procedure, as deviations can alter cerebral blood flow and metabolic rate, directly impacting ischemic lesion size.
9. **Animal Model Considerations:** This protocol was optimized for 3–4-month-old male C57BL/6 mice. The use of other strains, ages, or females may require re-optimization of coordinates. Young females are not recommended due to the influence of the estrogen cycle on lesion susceptibility.
10. **Post-operative Medications:** Do not administer anti-inflammatory drugs post-surgery, as they may interfere with the intended inflammatory response and confound the lesion outcomes.

***Note:*** Using a calibrated eye piece reticle can help monitor the volume injected into the brain.

### Problem 5

Weak antibody signal.

### Potential solution

A weak or absent antibody signal can primarily stem from two key aspects of tissue processing and pretreatment. The following solutions are recommended:

1. **Inadequate Antigen Retrieval:** The fixation process can mask target epitopes, making them inaccessible to antibodies. To resolve this:
  a. **Confirm Retrieval Buffer pH:** Ensure the use of the correct pH buffer (e.g., citrate buffer at pH 6.0 or Tris-EDTA buffer at pH 9.0) suitable for your specific target antigen.
  b. **Optimize Retrieval Conditions:** Precisely validate the incubation time and temperature when using a pressure cooker or water bath, as insufficient retrieval will weaken the signal, while over-retrieval can damage tissue morphology.
2. **Suboptimal Brain Section Preparation:** The method of sectioning can significantly impact antigen preservation.
  a. **Use Vibratome Sectioning Without Dehydration and OCT Embedding Steps:** For many antigens, particularly those sensitive to conformational changes, preparing free-floating sections using a vibratome is superior. This method avoids the extensive dehydration and freeze-thaw cycles inherent to cryostat sectioning, thereby better preserving the native structure of proteins and often resulting in a stronger antibody signal.
  b. **Section and Store the Slices in Anti-freeze in −20°C in a Timely Way.** Do not let the fixed brains or sectioned slices stay in 4°C for more than 7 days before sectioning or staining.

### Problem 6

Variable confocal intensity.

### Potential solution

Inconsistent signal intensity across images can compromise data quantification. This variability is frequently attributable to laser power fluctuations or optical limitations at greater imaging depths.

1. **Laser Instability:** To mitigate the effects of laser power drift, use identical imaging settings (including laser power, gain, and offset) for all samples within a comparative dataset. Regular laser warm-up and system calibration are recommended.
2. **Sample Depth Attenuation:** When imaging deep within a tissue section, signal intensity can diminish due to light scattering and absorption. To correct for this, perform a Z-axis calibration and consider using compensation settings to normalize intensity across the entire imaged volume.

## Resource availability

### Lead contact

Further information and requests for resources and reagents should be directed to and will be fulfilled by the lead contact, Min Tian (tianmin1111@gmail.com) or (mint@hkust-gz.ust.hk).

### Technical contact

Technical questions on executing this protocol should be directed to and will be answered by the technical contact, Min Tian (tianmin1111@gmail.com) or (mint@hkust-gz.ust.hk).

### Materials availability

This study did not generate any new unique reagents.

### Data and code availability

All raw and analyzed data are available upon request. No code is used in the current study.

## Acknowledgments

This project was supported by Outstanding Scholar under our Cross-disciplinary Research Funding for Visiting Scholars (CRFVS) Program at The Hong Kong University of Science and Technology (Guangzhou) (HKUST(GZ)), Ressler Family Foundation, NIH R37NS102185, The Dr. Miriam and Sheldon G. Adelson Medical Research Foundation, UCLA Eli and Edythe Broad Center of Regenerative Medicine and Stem Cell Research, including support from the Steffy Family Trust.

## Author contributions

M.T. and S.T. Carmichael initiated and drafted the manuscript. M.T. designed the study, conducted and analyzed most of the experiments. I.L. Llorente and M.T. designed the surgical procedure for vascular dementia model. M.T. is the technical contact, lead contact, and corresponding author. S.T. Carmichael is the senior and corresponding author.

## Declaration of interests

The authors declare no competing interests.

## Declaration of generative AI and AI-assisted technologies in the writing process

The authors used DeepSeek to check for grammar and style.
